# Editorial: Engineering plant-microbiomes to improve the health of economic crops

**DOI:** 10.3389/fpls.2026.1782531

**Published:** 2026-01-27

**Authors:** Zhen Wang, Ajay Kumar, Rachana Singh, Lucas Carvalho Basilio Azevedo, Manoj Kumar Solanki

**Affiliations:** 1 Sugarcane Research Institute, College of Agronomy and Biotechnology, Yunnan Agricultural University, Kunming,, China; 2 Amity Institute of Biotechnology, Amity University, Noida,, India; 3 Institute of Agricultural Sciences, Federal University of Uberlândia, Santa Monica,, Brazil; 4 Department of Life Sciences and Biological Sciences, IES University, Bhopal, Madhya Pradesh,, India

**Keywords:** economic crops, growth regulation, microbiome, plant health, stress adaptation

Economic crops are agricultural crops with specific economic uses and provide raw materials for industry. Together with food crops, they constitute the backbone of agricultural production, meeting daily dietary needs, supporting industrial systems, driving economic development, and optimizing agricultural structure, thereby forming a diversified, high-value sector of the global economy (Priyadarshan and Jain, 2022). Economic crops occupy a central position in global agricultural output value. In China, their output value accounts for approximately two-thirds of the total output value of the planting industry, significantly outweighing the contribution of food crops to increases in farmers’ income. Economic crops play an indispensable role in the global agricultural landscape by driving economic growth, ensuring food security, shaping trade patterns, and promoting technological innovation. The role of microbiome in plant health and growth is multidimensional and systematic, encompassing nutrient absorption, disease resistance, stress adaptation, and growth regulation (Noman et al., 2024). Through close symbiosis with plants, microbiomes become core partners in maintaining plant health. With continued advances in research, the precise application of microbial resources—such as synthetic microbial communities, microbial fertilizers, and biopesticides—is expected to become a key direction for sustainable agricultural development, providing strong support for achieving green development goals related to improving crop quality and yield (Wang et al., 2023). Building a healthy and stable plant microbiome is a core strategy for enhancing stress resistance and productivity in economic crops. Microbiomes play a key role in promoting nutrient uptake and maintaining plant health and therefore have substantial scientific and agronomic value for crop growth and yield.

This Research Topic focuses on advancing understanding of recent studies on interactions between economic crops and microbiomes. A total of 15 manuscripts were submitted, of which 9 articles were published, including 8 original research articles and 1 mini-review. These studies involve 7 crops—rice, *Rheum officinale*, citrus, maize, pepper, mango, and Aloe vera—and specifically describe the roles of *Botryosphaeria dothidea*, *Aureobasidium pullulans*, and *Burkholderia gladioli* in economic crops. The articles cover various biological and abiotic stress-related topics, such as plant nitrogen use efficiency, phosphorus solubilization and growth promotion, crop quality improvement, aphid infestation, and pathogen pathogenicity.

Bziuk et al. systematically explored the global distribution, ecological functions, and application potential of *Aureobasidium pullulans* within the frameworks of sustainable agriculture and the “One Health” concept, adopting a fungus with significant application value, from a novel microbiome-based perspective. Using the publicly available GlobalFungi database, the authors generated a macroscale global distribution map of *A. pullulans*. The analysis revealed a truly global distribution, with significantly higher detection rates in environments closely associated with human activities compared to natural ecosystems, highlighting a strong link to anthropogenic influence. These findings not only confirm the established value of *A. pullulans* as an effective biocontrol agent but also identity it as a natural core member of the plant microbiome. The results indicate that *A. pullulans* is not a transient colonizer but a resident symbiotic fungus with significant ecological influence. As a common component of the edible microbiome, *A. pullulans* may enter the human body through the food chain. Its potentially effects on the gut microbiome and human health was preliminarily explored, elevating its ecological significance to a “One Health” level that integrates plant, environmental, and human health. These findings provide a theoretical foundation for future in-depth development and application of *A. pullulans* in sustainable agriculture and health-related fields.

To explore how soybean green manure with different intercropping durations affects citrus fruit quality by altering soil physicochemical properties and microbial communities in acidified citrus orchards, Deng et al. established field experimental groups with varying intercropping durations. They systematically measured citrus fruit quality indicators and soil physicochemical properties and analyzed the community structures of soil bacteria and fungi using high-throughput sequencing. Through correlation network analysis, they revealed complex associations among intercropping duration, soil parameters, key microbial groups, and fruit quality. Soybean green manure significantly enhanced fruit quality, improved the soil environment, and reshaped the soil microbial community. Intercropping soybean green manure in citrus orchards is an effective ecological agricultural practice. Its core mechanism is the synergistic improvement of fruit quality through dual pathways: enhancement of soil physicochemical properties and regulation of soil microbial communities. This study provides a solid theoretical and practical basis for applying green manure intercropping in acidified orchard soils to support sustainable ecological restoration and improve agricultural production efficiency.

Lee et al. constructed a synthetic microbial community (SynCom) based on previous analyses of the tomato rhizosphere microbiome. They applied SynCom to pepper plants and compared its effects with those of individual strain inoculation, the chemical inducer benzothiadiazole (BTH), and a sterile water control. By analyzing volatile organic compounds (VOCs), and combining aphid infestation data, fruit yield measurements, and defense gene expression analyses, they comprehensively evaluated the effects of SynCom. SynCom significantly reduced aphid damage and increased crop yield. The microorganisms cooperatively produced the key volatile compound 1-nonanol, which could not be effectively produced by individual strains or partial combinations. Through exogenous addition experiments, treatment with 1 mM 1-nonanol reduced aphid numbers by 26–48% and activated expression of the pepper salicylic acid signaling pathway marker gene *CaPR1*, indicating a role in systemic resistance induction. This study demonstrates that microbial community cooperation can generate functional metabolites that cannot be synthesized by single strains. As a key signaling molecule, 1-nonanol indirectly surpasses pests by activating plant immunity. SynCom also maintained stability in complex field environments, providing a promising alternative to chemical pesticides and a theoretical foundation for designing efficient microbial fertilizers and biopesticides.

Wang et al. systematically examined the relationships among rhizosphere microbial community structure, soil physicochemical properties, and secondary metabolite accumulation in the cultivated medicinal plant *Rheum officinale* from three major production areas in China (ZB in Shaanxi, HB in Hubei, and CQ in Chongqing). *Rheum officinale* from the ZB region exhibited the highest content of rhein and catechin, whereas the CQ region was enriched in physcion. Microbial diversity followed the pattern ZB > HB > CQ, with bacteria dominated by Proteobacteria and fungi predominantly represented by Ascomycota. Soil properties, such as pH, moisture content, and zinc and copper ion concentrations, were closely correlated with microbial community structure. Increased pH and nutrient availability significantly promoted enrichment of beneficial bacterial groups such as Actinobacteria. Bacteria belonging to the order Rokubacteriales showed a significant positive correlation with anthraquinone accumulation, indicating that specific microbial groups may directly participate in regulating secondary metabolism through pathways such as nitrogen and phosphorus metabolism. This study elucidates the ecological mechanisms underlying geographical quality differentiation in rhubarb. Soil physicochemical properties indirectly regulate medicinal compound synthesis by shaping the rhizosphere microbiome, providing microbiome-level insights into the ecological cultivation of geographically indicated medicinal plants.

Guo et al. systematically investigated how *Burkholderia gladioli* DJB4-8, a highly efficient phosphate-solubilizing bacterium isolated from corn rhizosphere soil, promotes corn growth. By integrating multi-omics technologies with microbiology and plant physiology approaches, the study revealed the complete pathway by which the strain enhances crop growth through root colonization, regulation of endogenous plant hormones, and reprogramming of host metabolic pathways. Inoculation with DJB4–8 significantly promoted corn growth. After 40 days, plant height, root, stem, and leaf biomass, as well as photosynthetic rate, were significantly increased, and auxin content in the roots was elevated. At the molecular level, transcriptome analysis identified 303 differentially expressed genes, significantly enriched in pathways such as glutathione metabolism, plant hormone signal transduction, and phenylpropanoid biosynthesis. Metabolomic analysis further detected hundreds of differentially accumulated metabolites. Joint multi-omics analysis demonstrated that DJB4–8 synergistically regulates corn growth and development by activating core physiological processes such as plant hormone signaling, enhancing phenylpropane metabolism, and antioxidation in maize. This study elucidates the mechanism by which the phosphate-solubilizing bacterium DJB4–8 promotes corn growth via root colonization, physiological regulation, and metabolic reprogramming.

Umer et al. functionally characterized a serine carboxypeptidase gene, *Bd-SCP10*, from *Botryosphaeria dothidea*, a globally distributed pathogenic fungus affecting various fruit trees. The study aimed to uncover the enzyme’s role in fungal growth, pathogenicity, and stress tolerance, providing molecular targets for environmentally friendly disease management strategies. Bioinformatics analysis identified *Bd-SCP10* as a member of the S10 family in the *B. dothidea* genome. A gene knockout mutant (*ΔBd-SCP10*) was subsequently constructed, and a complementation strain (*CΔBd-SCP10*) was obtained through gene complementation. Systematic comparison of wild-type, mutant, and complementation strains under various conditions comprehensively evaluated the gene’s function. *Bd-SCP10* was confirmed as a pleiotropic gene regulating growth, pathogenicity, and stress response of *B. dothidea*. Its mechanism of action may include supplying nutrients for fungal growth by enzymaticallyhydrolyzing host proteins, modifying or degrading host defense proteins to suppress immune responses, and facilitating fungal colonization by maintaining cell wall integrity and redox balance. This study elucidates the core function of *Bd-SCP10* and establishes it as a promising molecular target.

Chandel et al. investigated the metabolic interactions between the medicinal plant *Aloe vera* and its rhizosphere-associated bacteria. *Aloe vera* selectively recruits beneficial bacteria from its rhizosphere and analyzes the mechanisms by which these bacteria promote plant growth and accumulation of medicinal active compounds. Using *Paenibacillus* sp. GLAU-BT2 and *Arthrobacter* sp. GLAU-BT16, separate and mixed inoculation treatments were established. The effects of these treatments on *Aloe vera* growth, leaf secondary metabolite content, and root exudate composition were evaluated in a pot experiment. Mixed bacterial inoculation significantly promoted plant growth and increased total phenolic, flavonoid, and flavonol content in leaves, as well as antioxidant activity, indicating that PGPR can enhance the medicinal quality of *Aloe vera*. Flavonoids were identified as key signaling molecules in rhizosphere metabolic interactions. *Aloe vera* attracts and shapes beneficial microbial communities by secreting flavonoids and other metabolites into its rhizosphere. These recruited PGPR (such as GLAU-BT2 and GLAU-BT16) colonize the roots and promote nutrient absorption by secreting plant hormones and solubilizing nutrients, thereby stimulating synthesis and accumulation of medicinal secondary metabolites. Some newly synthesized metabolites are then secreted back into the rhizosphere, further maintaining and enriching the beneficial bacterial community, forming a positive feedback loop. This study provides a theoretical basis and practical direction for improving the yield and active ingredient content of medicinal plants and developing sustainable agriculture through microbial regulation strategies.

Yang et al. investigated how different seasons (spring and autumn) and tree ages (10 and 28 years) affect the structure of arbuscular mycorrhizal fungi (AMF) communities in the rhizosphere soil of two mango orchards in Baise, Guangxi, China. They also conducted an in-depth analysis of the key environmental factors driving community changes. The study aimed to elucidate the core mechanisms influencing the assembly of mango AMF communities, providing a theoretical basis for promoting sustainable mango production through microbial approaches. Changes in tree age and season had no significant impact on the diversity or richness of mango rhizosphere AMF communities. Conversely, soil chemical properties—especially the contents of phosphorus (P), potassium (K), and their available forms (AP and AK)—were the dominant factors driving differences in AMF community structure. Compared to biological factors (tree age and season), abiotic factors (soil nutrients) were more crucial in shaping mango rhizosphere AMF communities. This study provides a novel microbial strategy for precision nutrient management and sustainable development in mango orchards.

Lao et al. conducted an in-depth study on the structure and function of endophytic bacterial communities within the seeds of rice varieties with different nitrogen use efficiency (NUE). Rice seeds harbor rich and diverse endophytic bacterial communities, and their structure varies significantly among varieties. Rare taxa are the main drivers of community diversity and differentiation, while core taxa are highly conserved across varieties, primarily composed of high-abundance taxa, and occupy central positions in the co-occurrence network, thereby maintaining community stability. Five representative bacterial strains successfully isolated from seeds exhibited various plant growth-promoting characteristics *in vitro*, including siderophore production, phosphate solubilization, and indole-3-acetic acid synthesis. All strains promoted rice growth, increased nitrogen accumulation, and enhanced NUE. However, their growth-promoting effects were co-regulated by strain identity and nitrogen supply levels. Under low nitrogen conditions, the growth-promoting effects of the strains were pronounced, highlighting their potential for application in nutrient-stressed environments. The endophytic bacterial community within rice seeds co-evolve with the host through strategies of innovation and adaptation driven by rare taxa, and stability and inheritance maintained by core taxa. These natural symbiotic bacterial communities, as microbial allies of the host, enhance host adaptability in low-nitrogen soils through synergistic mechanisms such as nitrogen fixation, regulation of nitrogen metabolism, and production of plant hormones.

Future research will focus on multi-dimensional analyses of plant-microbiome interaction networks. Synthetic biology and genome-editing technologies will facilitate the design of customized microbial communities, such as constructing SynComs with nitrogen fixation, phosphorus solubilization, and disease-resistance functions, enabling precise regulation of multiple effects from a single microbial inoculum. Simultaneously, strategies to enhance environmental adaptability are crucial. By utilizing directed evolution and metabolic engineering to modify microbial metabolic pathways, colonization stability under adverse conditions such as salinity, alkalinity, and drought can be improved. Furthermore, interdisciplinary collaboration will accelerate the exploration and industrialization of microbial resources, developing innovative solutions for agricultural carbon neutrality and food security, including living biopesticides based on endophytes and the construction of rhizosphere microbial seed banks.
